# Supplementary material for: Modeling glioblastoma heterogeneity as a dynamic network of cell states
Source: Mol Syst Biol. 2021 Sep 16;17(9):e10105. doi: 10.15252/msb.202010105 (PMC8444284; doi:10.15252/msb.202010105)
Supplement: Supplementary file 5 — Source Data for Figure 3 [file MSB-17-e10105-s001.zip › Figure3A_sourcedata/GSEA_3065/nosigdb_state6.GseaPreranked.1622718443993/LARSSON_STATE_4.html]

Details for gene set LARSSON\_STATE\_4[GSEA]

|  || Dataset | state6 |
| Phenotype | NoPhenotypeAvailable |
| Upregulated in class | na\_neg |
| GeneSet | LARSSON\_STATE\_4 |
| Enrichment Score (ES) | -0.53729355 |
| Normalized Enrichment Score (NES) | -2.1423407 |
| Nominal p-value | 0.0 |
| FDR q-value | 0.0 |
| FWER p-Value | 0.0 |
Table: GSEA Results Summary

  

Fig 1: Enrichment plot: LARSSON\_STATE\_4      
 Profile of the Running ES Score & Positions of GeneSet Members on the Rank Ordered List

  

| PROBE | GENE SYMBOL | GENE\_TITLE | RANK IN GENE LIST | RANK METRIC SCORE | RUNNING ES | CORE ENRICHMENT || 1 | PTMA |  |  | 94 | 0.216 | 0.0136 | No |
| 2 | TMSB15A |  |  | 253 | 0.161 | 0.0135 | No |
| 3 | HMGN2 |  |  | 679 | 0.108 | -0.0232 | No |
| 4 | LSM4 |  |  | 728 | 0.103 | -0.0171 | No |
| 5 | HIST1H1C |  |  | 768 | 0.101 | -0.0103 | No |
| 6 | H2AFV |  |  | 782 | 0.100 | -0.0006 | No |
| 7 | EZH2 |  |  | 940 | 0.089 | -0.0086 | No |
| 8 | MYBL2 |  |  | 991 | 0.086 | -0.0046 | No |
| 9 | NUCKS1 |  |  | 1066 | 0.082 | -0.0039 | No |
| 10 | CENPH |  |  | 1340 | 0.070 | -0.0275 | No |
| 11 | DEK |  |  | 1535 | 0.063 | -0.0427 | No |
| 12 | STMN1 |  |  | 1649 | 0.058 | -0.0492 | No |
| 13 | E2F1 |  |  | 2034 | 0.047 | -0.0880 | No |
| 14 | TUBA1B |  |  | 2087 | 0.045 | -0.0889 | No |
| 15 | PTTG1 |  |  | 2304 | 0.038 | -0.1094 | No |
| 16 | MND1 |  |  | 2472 | 0.034 | -0.1248 | No |
| 17 | RPA3 |  |  | 2563 | 0.032 | -0.1316 | No |
| 18 | LSM3 |  |  | 2673 | 0.029 | -0.1408 | No |
| 19 | TMPO |  |  | 2978 | 0.022 | -0.1732 | No |
| 20 | CKS1B |  |  | 2985 | 0.022 | -0.1714 | No |
| 21 | DTYMK |  |  | 3271 | 0.016 | -0.2024 | No |
| 22 | HNRNPD |  |  | 3359 | 0.014 | -0.2108 | No |
| 23 | DUT |  |  | 3596 | 0.009 | -0.2369 | No |
| 24 | RFC2 |  |  | 3618 | 0.009 | -0.2383 | No |
| 25 | HIST1H1E |  |  | 3635 | 0.008 | -0.2392 | No |
| 26 | PHF19 |  |  | 3654 | 0.008 | -0.2404 | No |
| 27 | ALYREF |  |  | 3755 | 0.006 | -0.2512 | No |
| 28 | MCM7 |  |  | 4435 | -0.008 | -0.3285 | No |
| 29 | SNRPD1 |  |  | 4477 | -0.008 | -0.3323 | No |
| 30 | MCM10 |  |  | 4638 | -0.011 | -0.3494 | No |
| 31 | UHRF1 |  |  | 4792 | -0.014 | -0.3654 | No |
| 32 | ANP32B |  |  | 4891 | -0.016 | -0.3748 | No |
| 33 | ASF1B |  |  | 4895 | -0.016 | -0.3733 | No |
| 34 | CENPM |  |  | 5089 | -0.020 | -0.3932 | No |
| 35 | GTSE1 |  |  | 5195 | -0.022 | -0.4028 | No |
| 36 | HIST1H1B |  |  | 5374 | -0.026 | -0.4204 | No |
| 37 | H2AFX |  |  | 5451 | -0.027 | -0.4260 | No |
| 38 | LMNB1 |  |  | 5545 | -0.029 | -0.4334 | No |
| 39 | NUDT1 |  |  | 6071 | -0.040 | -0.4894 | No |
| 40 | H2AFZ |  |  | 6079 | -0.040 | -0.4857 | No |
| 41 | CENPN |  |  | 6194 | -0.042 | -0.4940 | No |
| 42 | CDKN3 |  |  | 6291 | -0.044 | -0.5000 | No |
| 43 | RANBP1 |  |  | 6309 | -0.045 | -0.4970 | No |
| 44 | CDC45 |  |  | 6326 | -0.045 | -0.4937 | No |
| 45 | PCLAF |  |  | 6365 | -0.046 | -0.4929 | No |
| 46 | GINS2 |  |  | 6461 | -0.049 | -0.4983 | No |
| 47 | FANCI |  |  | 6473 | -0.049 | -0.4940 | No |
| 48 | MKI67 |  |  | 6750 | -0.057 | -0.5193 | No |
| 49 | MCM5 |  |  | 6765 | -0.058 | -0.5144 | No |
| 50 | TUBB |  |  | 6820 | -0.059 | -0.5139 | No |
| 51 | BIRC5 |  |  | 7024 | -0.066 | -0.5299 | Yes |
| 52 | SIVA1 |  |  | 7054 | -0.066 | -0.5258 | Yes |
| 53 | HIST1H1D |  |  | 7093 | -0.068 | -0.5225 | Yes |
| 54 | HMGB1 |  |  | 7186 | -0.071 | -0.5252 | Yes |
| 55 | RAN |  |  | 7221 | -0.072 | -0.5210 | Yes |
| 56 | RAD51AP1 |  |  | 7236 | -0.072 | -0.5145 | Yes |
| 57 | ZWINT |  |  | 7370 | -0.077 | -0.5211 | Yes |
| 58 | TOP2A |  |  | 7430 | -0.079 | -0.5191 | Yes |
| 59 | RRM1 |  |  | 7491 | -0.081 | -0.5169 | Yes |
| 60 | TK1 |  |  | 7499 | -0.081 | -0.5085 | Yes |
| 61 | PIMREG |  |  | 7526 | -0.083 | -0.5022 | Yes |
| 62 | MCM3 |  |  | 7597 | -0.086 | -0.5006 | Yes |
| 63 | SGO2 |  |  | 7632 | -0.087 | -0.4947 | Yes |
| 64 | DBF4 |  |  | 7639 | -0.088 | -0.4855 | Yes |
| 65 | HIST1H4C |  |  | 7641 | -0.088 | -0.4758 | Yes |
| 66 | GMNN |  |  | 7651 | -0.088 | -0.4669 | Yes |
| 67 | LSM5 |  |  | 7653 | -0.088 | -0.4570 | Yes |
| 68 | ESCO2 |  |  | 7778 | -0.094 | -0.4607 | Yes |
| 69 | UBE2S |  |  | 7818 | -0.097 | -0.4543 | Yes |
| 70 | MCM4 |  |  | 7900 | -0.101 | -0.4522 | Yes |
| 71 | PBK |  |  | 7905 | -0.101 | -0.4412 | Yes |
| 72 | CCNB1 |  |  | 7958 | -0.104 | -0.4355 | Yes |
| 73 | CHAF1A |  |  | 7967 | -0.105 | -0.4246 | Yes |
| 74 | RRM2 |  |  | 8001 | -0.107 | -0.4163 | Yes |
| 75 | KPNA2 |  |  | 8045 | -0.110 | -0.4089 | Yes |
| 76 | PCNA |  |  | 8061 | -0.111 | -0.3981 | Yes |
| 77 | CKS2 |  |  | 8077 | -0.112 | -0.3873 | Yes |
| 78 | KIF15 |  |  | 8228 | -0.124 | -0.3906 | Yes |
| 79 | TYMS |  |  | 8287 | -0.131 | -0.3825 | Yes |
| 80 | KIF4A |  |  | 8300 | -0.132 | -0.3690 | Yes |
| 81 | FBXO5 |  |  | 8310 | -0.133 | -0.3551 | Yes |
| 82 | HIST1H1A |  |  | 8345 | -0.137 | -0.3435 | Yes |
| 83 | CDK1 |  |  | 8351 | -0.139 | -0.3284 | Yes |
| 84 | ATAD2 |  |  | 8359 | -0.141 | -0.3134 | Yes |
| 85 | TPX2 |  |  | 8370 | -0.143 | -0.2985 | Yes |
| 86 | HMGB2 |  |  | 8388 | -0.145 | -0.2841 | Yes |
| 87 | NCAPG |  |  | 8390 | -0.145 | -0.2679 | Yes |
| 88 | CENPU |  |  | 8403 | -0.147 | -0.2527 | Yes |
| 89 | MAD2L1 |  |  | 8427 | -0.150 | -0.2386 | Yes |
| 90 | CLSPN |  |  | 8440 | -0.151 | -0.2229 | Yes |
| 91 | ORC6 |  |  | 8482 | -0.157 | -0.2099 | Yes |
| 92 | UBE2T |  |  | 8489 | -0.159 | -0.1927 | Yes |
| 93 | NUSAP1 |  |  | 8523 | -0.167 | -0.1778 | Yes |
| 94 | SGO1 |  |  | 8571 | -0.178 | -0.1631 | Yes |
| 95 | DHFR |  |  | 8590 | -0.186 | -0.1442 | Yes |
| 96 | SMC2 |  |  | 8658 | -0.216 | -0.1277 | Yes |
| 97 | BRCA1 |  |  | 8659 | -0.217 | -0.1032 | Yes |
| 98 | CENPK |  |  | 8666 | -0.220 | -0.0792 | Yes |
| 99 | CENPF |  |  | 8703 | -0.240 | -0.0563 | Yes |
| 100 | UBE2C |  |  | 8748 | -0.286 | -0.0292 | Yes |
| 101 | SMC4 |  |  | 8764 | -0.307 | 0.0037 | Yes |
Table: GSEA details [plain text format]

  

Fig 2: LARSSON\_STATE\_4: Random ES distribution      
 Gene set null distribution of ES for **LARSSON\_STATE\_4**
